# Soft Tissue Chondroma: A Possible Diagnosis of Single-Digit Nail Clubbing

**DOI:** 10.1097/DSS.0000000000003295

**Published:** 2021-11-09

**Authors:** Siliang Xue, Yusha Chen, Eckart Haneke

**Affiliations:** * Department of Dermatovenereology, Sichuan University West China Hospital, Chengdu, China; † Department of Dermatology, Inselspital, Bern University Hospital, Bern, Switzerland

## Abstract

Supplemental Digital Content is Available in the Text.

Soft tissue chondroma (STC) is a benign, slow-growing cartilaginous tumor characterized by well-demarcated nodule(s) of mature cartilage unattached to the adjacent bone. It commonly occurs in middle-aged people with a predilection to the distal extremities but rarely involving the subungual area. It has not been reported to induce nail clubbing.^1,2^

We describe an unusual case of single-digit nail clubbing in a rare location of STC and briefly discuss its differential diagnosis. Patients developing single-digit nail clubbing should alert the dermatologist to consider the possibility of STC.

A 58-year-old man presented with a clubbed nail of his left index finger without any previous trauma event in the past 10 years. The single-digit clubbing had been ignored by him over the past decade because he had no symptoms. Because the clubbing of his nail gradually enlarged and eventually severely deformed the fingertip resembling a ball, he visited our institution. Dermatologic examination revealed a clubbed nail extending from the distal interphalangeal (DIP) joint to the hyponychium with an increased transverse and longitudinal curvature (Figure 1A). The suspected mass under the nail was hard, elastic, and immobile on palpation and without any signs of inflammation. Of note, there was no movement restriction of the DIP joint. Laboratory test results were normal. Plain radiographs showed a soft tissue tumor shadow, located subcutaneously on the dorsal aspect of the distal phalanx of his index finger, extending from the distal dorsal DIP joint through the subungual region up to the hyponychium (Figure 1B). The tumor had no continuity with the underlying bone. In addition, there were no destructive changes in the distal phalanx and no periosteal reaction. Ultrasound examination exhibited an ovoid, well-defined, heterogenous hypoechoic nodule but without vascularity or continuity to the nearby bone (See Figure S1, **Supplemental Digital Content**, http://links.lww.com/DSS/A941). Based on these findings, the mass was believed to be a cartilaginous tumor, and excision was performed. After nail avulsion and incision of the extremely thin nail bed over the mass, a white solid tumor appeared. There was no adhesion to the periosteum, and it could be easily separated from the surrounding tissue. Its diameter was about 20 mm (Figure 1C). The tumor was fragile and tended to break when using tweezers to hold it. Histopathology of the tumor revealed mature hyaline cartilage without nuclear pleomorphism (Figure 1D). Thus, it was diagnosed as a STC involving the nail unit. Healing was uneventful, and his nail grew by about a third with slightly uneven nail plate during the 6-month follow-up period.

**Figure 1.**
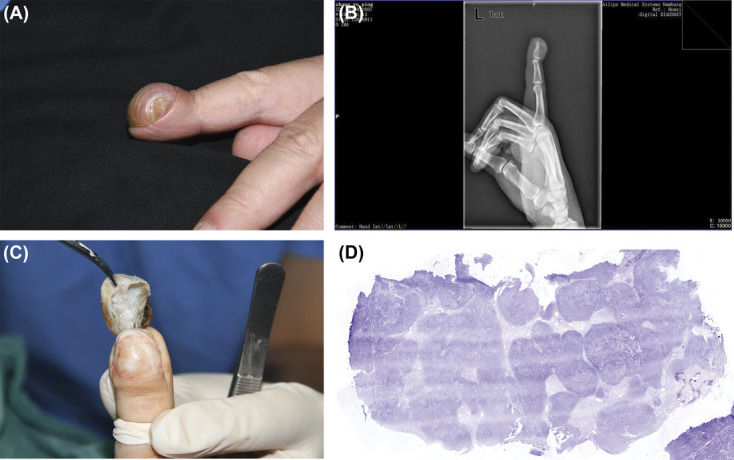
(A) The nail had an increased transverse and longitudinal curvature. (B) Plain radiographs demonstrated a soft tissue tumor shadow. (C) A white solid tumor appeared after removing the nail plate and extremely thin nail bed. (D) Histopathological image of the tumor (H&E, scanned at 20 magnification).

Soft tissue chondroma is a rare, benign, cartilaginous tumor that is believed to derive from fibroblasts and unattached to the underlying bone. It mainly occurs on the hands or feet of individuals aged 30 to 60 years and rarely involves the subungual part and commonly appears as an asymptomatic slow-growing mass. Over time, the patients complain of symptoms such as local tenderness and pain on action. Swelling of the distal phalanx of the middle finger with progressive distortion of the nail was reported. Histopathology revealed fairly normal mature hyaline cartilage.^1,2^

Clinically, STC is frequently misdiagnosed because of partial overlap manifestations with soft tissue osteochondroma (STO), juxtacortical (periosteal) chondroma, subungual exostoses, enchondroma, superficial acral fibromyxoma, subungual lipoma, etc., particularly with STO that has been ambiguously classified as STC but actually is a different entity. Radiology of STO shows a calcified soft tissue mass with central mature ossification while histopathology exhibits areas of endochondral ossification with mature trabecular bone. The juxtacortical chondroma is a slow-growing cartilaginous lesion, which arises adjacent to the cortex beneath the periosteum. Subungual exostoses are outgrowths of normal bone or calcified cartilaginous remains; the histopathology shows formation of trabecular bone in the base of the lesion covered by a fibrocartilaginous layer. Enchondroma is frequently asymptomatic but may enlarge and become a painful tumor that expands the tip of the finger; radiologically, it shows cortical thinning and enlargement, usually due to unilateral bone cortex defects, but some had both volar and dorsal bone cortex defects. Superficial acral fibromyxoma is a slow-growing, solitary, erythematous, elastic tumor with a striking predilection for the subungual and periungual region of the hands and feet. Histopathologically, the lesions are well-circumscribed but not encapsulated dermal nodules composed of stellate and spindle cells, arranged in a myxoid collagenous matrix. Lipomas can occur in the distal phalanx of the thumb causing a tender and painful swelling and scalloping of the distal bony phalanx; some of them also present single-digit nail clubbing. Histopathologically, it resembled nevus lipomatosus superficialis.^3–5^

This individual presenting with an infrequent single-digit nail clubbing due to an STC highlights the adaptive deformability of the nail. Distinction of STC from other subungual neoplasms can be supported by an asymptomatic slow-growing mass, no continuity with the neighboring bone, and mature hyaline cartilage on histology.

## Supplementary Material

SUPPLEMENTARY MATERIAL
