# Supplementary figures and images for: Soft Tissue Chondroma: A Possible Diagnosis of Single-Digit Nail Clubbing
Source: Dermatol Surg. 2021 Nov 9;48(1):143–4. doi: 10.1097/DSS.0000000000003295 (PMC8667792; doi:10.1097/DSS.0000000000003295)

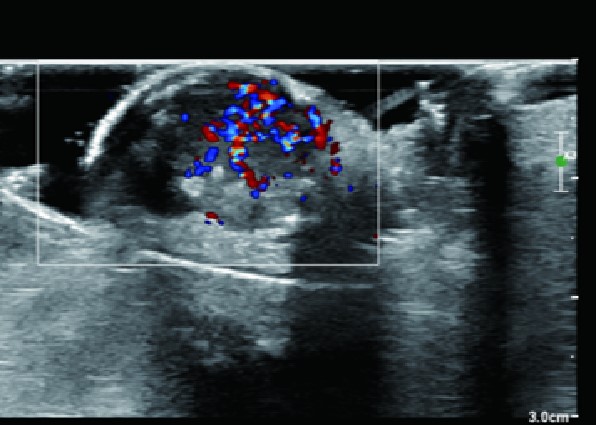


**Figure S1**

Supplement: SUPPLEMENTARY MATERIAL [file ds-48-143-s001.docx]
